# Valorization of Aquaculture By-Products of Salmonids to Produce Enzymatic Hydrolysates: Process Optimization, Chemical Characterization and Evaluation of Bioactives

**DOI:** 10.3390/md17120676

**Published:** 2019-11-30

**Authors:** José Antonio Vázquez, Carmen G. Sotelo, Noelia Sanz, Ricardo I. Pérez-Martín, Isabel Rodríguez-Amado, Jesus Valcarcel

**Affiliations:** 1 Grupo de Biotecnología y Bioprocesos Marinos, Instituto de Investigaciones Marinas (IIM-CSIC), C/Eduardo Cabello, 6, CP 36208 Vigo, Galicia, Spain; carmen@iim.csic.es (C.G.S.); nsanz@iim.csic.es (N.S.); ricardo@iim.csic.es (R.I.P.-M.); jvalcarcel@iim.csic.es (J.V.); 2 Laboratorio de Reciclado y Valorización de Materiales Residuales (REVAL), Instituto de Investigaciones Marinas (IIM-CSIC), CP 36208 Vigo, Galicia, Spain; 3 Laboratorio de Bioquímica de Alimentos, Instituto de Investigaciones Marinas (IIM-CSIC), CP 36208 Vigo, Galicia, Spain; 4 Department of Life Sciences of the International Iberian Nanotechnology Laboratory (INL), Avenida Mestre José Veiga, Braga 4715-330, Portugal; sabelara@uvigo.es

**Keywords:** salmonids by-products valorization, fish protein hydrolysates, bioactives, mathematical optimization

## Abstract

In the present manuscript, various by-products (heads, trimmings, and frames) generated from salmonids (rainbow trout and salmon) processing were evaluated as substrates for the production of fish protein hydrolysates (FPHs), potentially adequate as protein ingredients of aquaculture feeds. Initially, enzymatic conditions of hydrolysis were optimized using second order rotatable designs and multivariable statistical analysis. The optimal conditions for the Alcalase hydrolysis of heads were 0.1% (v/w) of enzyme concentration, pH 8.27, 56.2°C, ratio (Solid:Liquid = 1:1), 3 h of hydrolysis, and agitation of 200 rpm for rainbow trout and 0.2% (v/w) of enzyme, pH 8.98, 64.2 °C, 200 rpm, 3 h of hydrolysis, and S:L = 1:1 for salmon. These conditions obtained at 100 mL-reactor scale were then validated at 5L-reactor scale. The hydrolytic capacity of Alcalase and the protein quality of FPHs were excellent in terms of digestion of wastes (V_dig_ > 84%), high degrees of hydrolysis (*H_m_* > 30%), high concentration of soluble protein (Prs > 48 g/L), good balance of amino acids, and almost full *in vitro* digestibility (Dig > 93%). Fish oils were recovered from wastes jointly with FPHs and bioactive properties of hydrolysates (antioxidant and antihypertensive) were also determined. The salmon FPHs from trimmings + frames (TF) showed the higher protein content in comparison to the rest of FPHs from salmonids. Average molecular weights of salmonid-FPHs ranged from 1.4 to 2.0 kDa and the peptide sizes distribution indicated that hydrolysates of rainbow trout heads and salmon TF led to the highest percentages of small peptides (0–500 Da).

## 1. Introduction

The production of aquaculture fish around the world achieved 80 million tons in 2016, supposing 48% of the total fish captured, transformed, and marketed [1]. Salmonids production, rainbow trout (*Oncorhynchus mykiss*) and mainly Atlantic salmon (*Salmon salar*), is the most important in economic and volume terms in the European fish farming system. More than 1.78 MTm of both were produced in 2016 generating more than € 15 billion [1]. Although in southern Europe the most common way to market salmonids is as complete individuals, in the rest of the continent the tendency is to market them in the form of fillets (fresh, frozen, cured, etc.) or other presentations that require deheading, gutting, and filleting steps. Thus, huge amounts of by-products (about 35–45% of the total weight of salmonids) are generated in processing plants, mainly heads, trimmings, viscera, and frames that have to be managed efficiently to reduce environmental health problems and to improve the sustainability of such farming productions [2,3].

The most habitual utilization of salmonid wastes is based on the development of acid silage [3], fertilizers, or the joint production of fishmeal and oils [4,5]. In the first case, the silage is a low-added value product with limited applications [1]. In the last one, oil and fishmeal are not very sustainable productions when the fishmeal equipments are far from fishing ports or aquaculture plants and close to villages and cities due to the environmental impact (odors, air pollution, water consumption, etc.) that this production generates. This process leads to the coagulation of the protein and its separation from the oil but the level of valorization achieved by biomass is low-medium. In this context, valorization processes focused on the enzyme proteolysis to generate fish protein hydrolysates (FPHs), including the recovery of bioactive compounds and essential nutrients [6,7], maybe a feasible and adequate protocol to efficiently upgrade aquaculture wastes. Enzymatic hydrolysis of fish wastes involves a highly controllable and reproducible method for the separation of bones, oils, and peptide fractions from complex matrices. Several fish species, including from farm origin, employing different proteases and conditions of hydrolysis have been studied in the last two decades [2,8,9,10]. The functional capacity of FPHs in terms of antihypertensive, antioxidant, antiproliferative, antimicrobial, etc., *in vitro* bioactivities, is one of the most valuable properties of these bioproduction [11,12,13,14]. Additionally, since hydrolysates are composed of soluble proteins, peptides, and free amino acids, they are an excellent ingredient of aquaculture feeds and pet-food diets in substitution of the conventional fishmeals, improving generally the effectiveness of feeds and diets to support fish and animal healthy growths [15,16,17,18]. 

However, complete studies of production of enzymatic hydrolysates from salmonid wastes including optimization of proteolysis conditions, analysis of kinetic hydrolysis, chemical characterization of all products obtained, bioactivities, and peptides size distribution are practically non-existent. Therefore, the aims of this work are (1) optimization of the experimental conditions to produce FPHs, using Alcalase, of salmonid by-products (heads of salmon and rainbow trout) by response surface methodology (RSM), (2) mathematical analysis of hydrolysis kinetics by Weibull equation, (3) chemical characterization of products obtained from salmonid hydrolysis, (4) identification of average molecular weights and peptide size distribution of the hydrolysates, and (5) determination of two bioactives (antioxidant and antihypertensive) from FPHs. 

## 2. Results and Discussion

The proximate composition of salmonids raw materials is summarized in Table 1 (RT_H: heads of rainbow trout, RT_TF: trimmings + frames of rainbow trout, S_H: heads of salmon, S_TF: trimmings + frames of salmon). The moisture of these samples ranged between 66% and 70% and the organic matter was higher in salmon than rainbow trout and also superior in trimmings + frames compared to heads by-products. TF showed a greater amount of proteins and a lower level of total lipids. S_H was the fattest by-product (Table 1). 

### 2.1. Optimization of Salmonid By-Products Hydrolysis

Optimization of salmonid heads hydrolysis was studied according to the factorial designs summarized in Table S1 (supplementary material) using a pH-stat system (100 mL reactor). Alcalase 2.4 L was chosen as biocatalyst due to its excellent capacity of proteolysis when it was applied to several marine substrates as squid pens, fish cartilages, crustacean shells, and other fish tissues and by-products [19,20,21,22]. A two-variable factorial design was executed in anticipation that no interactions among pH, T, r (S:L), and enzyme concentration were expected as it was reported by Liaset et al. [3]. The effects of those last independent variables were individually studied after optimization experiments.

**Figure 1:**
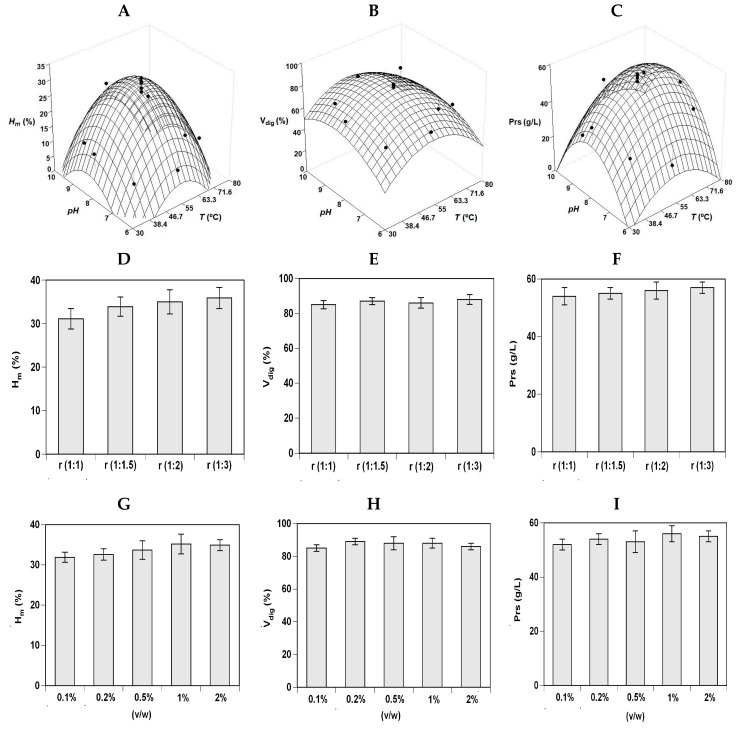
Optimization studies of RT_H hydrolysis by Alcalase. Experimental data and theoretical surfaces showing the combined influence of pH and *T* on *H_m_* (**A**), V_dig_ (**B**), and Prs (**C**) as defined in Table 3 and Table 4. (**D**) Individual influence of Alcalase concentration on *H_m_*. (**E**) Individual influence of Alcalase concentration on V_dig_. (**F**) Individual influence of Alcalase concentration on Prs. (**G**) Individual influence of S:L ratio on *H_m_*. (**H**) Individual influence of S:L ratio on V_dig_. (**I**) Individual influence of S:L ratio on Prs. Error bars show the intervals of confidence for *n* = 2 (replicates of independent hydrolysates) and α = 0.05.

**Figure 2:**
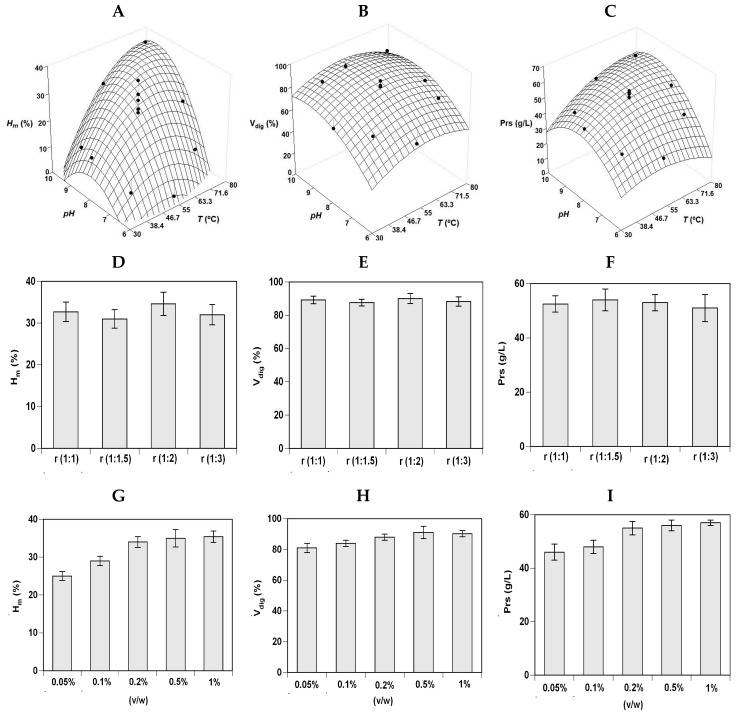
Optimization studies of S_H hydrolysis by Alcalase. Experimental data and theoretical surfaces showing the combined influence of pH and *T* on *H_m_* (**A**), V_dig_ (**B**), and Prs (**C**) as defined in Table 3 and Table 4. (**D**) Individual influence of Alcalase concentration on *H_m_*. (**E**) Individual influence of Alcalase concentration on V_dig_. (**F**) Individual influence of Alcalase concentration on Prs. (**G**) Individual influence of S:L ratio on *H_m_*. (**H**) Individual influence of S:L ratio on V_dig_. (**I**) Individual influence of S:L ratio on Prs. Error bars show the intervals of confidence for *n* = 2 (replicates of independent hydrolysates) and α = 0.05.

Figure 1 and Figure 2 (A, B, and C plots) show the experimental data and the corresponding theoretical surfaces for the three responses of RT_H and S_H hydrolysis. Empirical equations were calculated from factorial data and optima values determined for each case studied (Table 2). From a statistical point of view, the degrees of explicability of the polynomials (concordance among simulated and experimental data) were ranging from 76% to 91% for the three responses and the two substrates evaluated. The robustness of the polynomials was also statistically validated by Fisher tests (F1 and F2) (data not shown). Average data of *pH_opt_* and *T_opt_* were 8.27 and 56.2 °C for RT_H and 8.98 and 64.2 °C for S_H. 

Based on these conditions, the concentration of protease and the S:L ratio that maximizes the production of FPHs was subsequently evaluated in one-factor-at-a-time method (Figure 1D–I). For RT_H, the values of *H_m_* increased at higher (S:L) but no significant differences were found among ratios for the responses V_dig_ and Prs (*p* > 0.05). The effect of Alcalase concentration followed a similar trend: higher *H_m_* value at larger enzyme added but similar response for V_dig_ and Prs results. Thus, (1:1) ratio and 0.1% (v/w) of commercial protease were selected as the most adequate conditions to digest the trout by-products, therefore, reducing the costs of the hydrolysis stage.

In a similar way, the single effect of solid:liquid ratio and Alcalase concentration on S_H hydrolysis were tested maintaining constant the average values of pH_opt_ and T_opt_ previously defined. The three responses are displayed in Figure 2 (D–I plots) indicating the lack of significant differences between the ratios studied. All responses from hydrolysis (H_m_, V_dig_ and Prs) rose with the increase in the protease used up to an Alcalase concentration of 0.2% v/w. Thus, ratio of (1:1) and 0.2% of enzyme were the conditions chosen for carrying out the hydrolysis of salmon wastes to produce aquaculture feed ingredients.

In summary, the optimal conditions obtained for salmonids hydrolysis were (pH and T calculated as the average of the values indicated in Table 2): 1) Alcalase 0.1%, pH 8.27, 56.2 °C, 200 rpm, 3 h of hydrolysis, and S:L = 1:1 for RT_H and RT_TF; 2) Alcalase 0.2%, pH 8.98, 64.2 °C, 200 rpm, 3 h of hydrolysis, and S:L = 1:1 for S_H and S_TF. Optimal conditions for RT were similar to those found for skin salmon treated with Alcalase (55.3 °C and pH 8.39) but needing much less concentration of enzyme (0.1% *vs.* 2.5%) [23].

### 2.2. Production and Chemical Composition of FPHs

**Figure 3:**
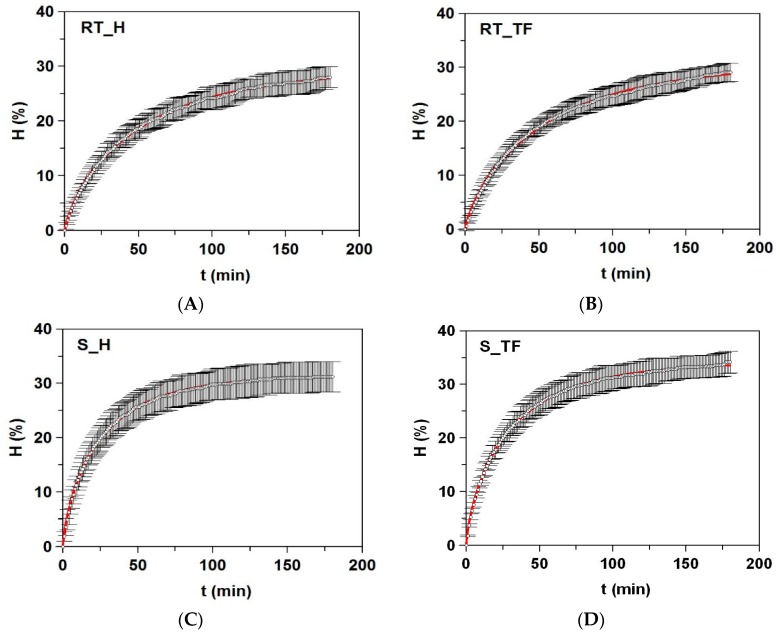
Degree of hydrolysis (*H*) of salmonids wastes by Alcalase: (**A**) RT_H: heads of rainbow trout; (**B**) RT_TF: trimmings and frames of rainbow trout; (**C**) S_H: heads of salmon, and (**D**) S_TF: trimmings and frames of salmon. Weibull equation (continuous line) modeled the time-course of hydrolysis degrees (symbols). Error bars show the intervals of confidence for *n* = 18–20 (replicates of independent hydrolysates) and α = 0.05.

The next step was to validate these optimized conditions in a high-scale performance. Thus, hydrolysates (among 18–20 independent batches) were run in a 5L-pH-stat reactor with 2 kg of ground raw material. In all cases, the kinetics of hydrolysis (*H*) were accurately described by Weibull equation (Table 3, Figure 3). The agreement between experimental and simulated data was total (R^2^>0.999) and the statistical feasibility of equation was also confirmed by F-Fisher test (*p*<0.005). The maximum degrees of hydrolysis (*H_m_*) were slightly greater in salmon by-products, whereas the maximum rates of hydrolysis were slower on trout wastes. In addition, these numerical values of parameters were similar to those obtained in 100 mL-reactor and reported in Table 3 and Figure 1 and Figure 2. In all reports about the production of salmonid FPHs, the mathematical modeling of proteolytic kinetics was unexplored. Taking into account the published data of H (%) at the end of Alcalase treatment, our values of *H_m_* (Table 3) were always higher or slightly higher than those obtained for hydrolysates of salmon head (17%), salmon frames (27%), and trout roe (28%) [9,24,25]. Using soluble proteins extracted by CaCl_2_-citric treatment of a mixture of RT by-products (heads, frames, and viscera) as substrate, the value of H was of 42% for a 3-h Alcalase hydrolysate [26]. In addition to the type of starting material, the concentration of enzyme in that work was 50 times higher than that used in the present experiments.

The processing of salmonid hydrolysates was performed using the protocol shown in Supplementary material (Figure S1). Figure S2 (Supplementary material) presents different photographs related to the production of FPHs and other resulting products. For example, one by-product of the hydrolysates production is bones, the percentage recovered in filters after hydrolysis was around 9–12% (w/w of initial substrate) and the yield is higher in salmon than in trout (Table 4). These results are lower than the amount of bones recovered from heads of different fish discards (red scorpionfish, blue whiting, mackerel, megrim, boardfish, etc.) after enzymatic digestion [10,27]. Besides, the recovered bones did not present a significant amount of residual muscle or organic material. 

Oil was also separated from FPHs; in this case, the yield was around 9–11% (v/w of initial substrate) and the highest volume of fish oil was found in S_H. In this context, heads from red salmon yielded the same volume of oil (10.6%) after Alcalase hydrolysis and mechanical separation [4]. The composition in fatty acids of the oils was summarized in Table S2 (Supplementary material). Oleic acid (>50%) and linoleic acid (>12%) were the main fatty acids present in oils and the amount of docosahexaenoic acid (DHA) and eicosapentaenoic acid (EPA), the most relevant from their biological properties, did not exceed 3%. This percentage contrasts with the levels of these omega-3 (more than 26%) in oils recovered from salmon frames hydrolysates in an article published several years ago [3]. This huge difference could be attributed to the current salmon diets, in which vegetal oils and meals have largely replaced the more expensive oils and meals from fish origin. Blanchet et al. [28] studied the differences between fatty acids composition in wild and farmed salmonids (salmon and trout). In all cases, omega-3 content in oils present here were lower than reported in the mentioned publication. In addition, our omega-3/omega-6 ratios for salmonid oils recovered after Alcalase hydrolysis were lower than or equal to 0.5, revealing their low potential as ingredients for nutraceutical applications [29]. 

The ability of Alcalase for the digestion (V_dig_) of raw materials was always higher than 84% (RT_TF), with a maximum value of 90% detected in S_H. The values of V_dig_ were significantly superior in H than TF (*p* < 0.05). The levels reported here were in agreement with those observed in the production of hydrolysates of fish discards [10,27]. Depending on the method used for the quantification of proteins, the levels of protein material present in farmed FPHs ranged 48–69 g/L, 53–71 g/L, and 52–73 g/L for Prs, Pr-tN, and Pr (Σaa), respectively. TF substrates led to a larger concentration of protein in comparison to heads. Regarding species, salmon FPHs showed higher protein concentration than those obtained with trout. The *In vitro* digestibility (Dig) of FPHs was excellent, in all cases, it has been found values higher than 92% without significant differences between FPHs (*p* > 0.05).

Regarding amino acid content in FPHs, the main ones are glutamic acid (Glu), aspartic acid (Asp), and glycine (Gly), but all the essential amino acids are included in the salmonid hydrolysates produced here (Table S3, Supplementary material). In all cases, the essential amino acid content was higher (value of TEAA/TAA as percentage) than recommended for human adults and infants [30,31]. Similar percentages to our outcomes were observed for enzymatic hydrolysates of rainbow trout frames and roes generated by Alcalase [14,25], and salmon frames catalyzed with Protamex [3] but inferior when Papain was applied to identical salmon wastes [9]. Nevertheless, the data of TEAA/TAA for salmon viscera hydrolysates [32] were higher than here reported for other by-products of salmonids (46% *vs.* 33–37%). Protein, amino acid contents and digestibilities shown in Table S3 and Table 4 were in concordance with the chemical, functional, and nutritional properties necessary for their utilization in animal feed [15,16,18]. Additionally, the values of total sugars, from 1.2 to 1.5 g/L, were very similar in the four hydrolysates of salmonids. 

**Figure 4:**
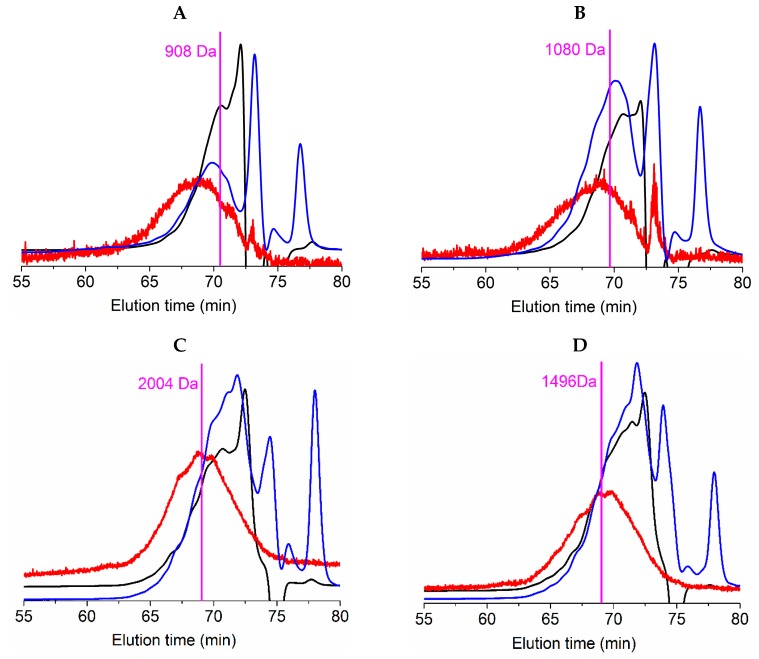
Gel permeation chromatography (GPC) eluograms of fish protein hydrolysates (FPHs) (**A**: RT_H, **B**: RT_TF, **C**: S_H, and **D**: S_TF). Black line: refractive index; blue line: UV (232 nm); red line: right angle light scattering; vertical lines: number average molecular weight (Mn).

Average molecular weight (Mw) of protein fraction in salmonid FPHs were (Table 5): 1944 ± 264 Da (index of polydispersity, PD: 2.11) for RT_H, 1682 ± 65 Da (PD: 1.58) for RT_TF, 1945 ± 136 Da (PD: 1.57) for S_H, and 1442 ± 51 Da (PD: 1.53) for S_TF. In the case of the number average molecular weight (Mn) of salmonid FPHs, the results obtained from gel permeation chromatography (GPC) were: 920 ± 110 Da for RT_H, 1067 ± 152 Da for RT_TF, 1235 ± 91 Da (PD: 1.57) for S_H, and 944 ± 40 Da (PD: 1.53) for S_TF. A representation of GPC-profiles of such peptides distribution from FPHs is displayed in Figure 4 and is also indicated in Table 5. 

The distribution of peptide sizes (as percentage), quantified by means of two types of chromatographic procedures, are also summarized in Table 5 and represented in Figure S3 (Supplementary material). As described for Mn and Mw, S_H hydrolysates produced peptides with the highest distribution of sizes (67% of peptides > 1 kDa) followed by RT_TF (58%). The hydrolysate with the greatest percentage of small peptides (0–200 Da) was RT_H (13.7%). Nikoo and coauthors [26] produced a FPH with greater percentage of low peptides (0–0.2 kDa: 40%, 0.2–0.5 kDa: 24% and 0.5–1 kDa: 14%) employing more amount of enzyme on protein chemically solubilized from different rainbow trout by-products. For its part, salmon head digested by Alcalase for 2 h led to a higher size distribution (100% of peptides above 1.4 kDa) and, as mentioned, a lower degree of hydrolysis (17%) [24]. 

### 2.3. In Vitro Bioactivities of Hydrolysates from Salmonids By-Products

The data of activities (antioxidant, AO and antihypertensive, AH) for FPHs samples are included in Table 6. The hydrolysates of TF from both fish showed significant larger 1,1-Diphenyl-2-picryhydrazyl (DPPH) activities than those recovered from salmonid heads (*p* < 0.05). Nevertheless, S_H led to the significant lowest data of ABTS (2,2′-azinobis-(3-ethyl-benzothiazoline-6-sulphonic acid) and Crocin (*p* < 0.05). In general, the numerical response of DPPH method, Crocin protocol was never employed to analyze salmonid samples, was not sufficiently attractive when are compared with samples of Alcalase hydrolysates from trout roe [25] and for soluble proteins obtained by chemical solubilization of a mixture of RT by-products [33]. However, enzymatic hydrolysates from pectoral fin and collagen skin of salmon showed similar % of DPPH than those listed in Table 6 [34,35]. Based on data from FPHs of other marine wastes, our DPPH activities were greater than hydrolysates of hake [36] and lower than samples of cuttlefish, herring, or croaker [37,38,39]. In relation to ABTS determinations, Idowu et al. [9] reported more than 10 times activity (688 μmol trolox/g) for frames of salmon digested with 3% of Alcalase for 3 h than that found in salmonid samples described here. It is important to mention that in those samples of frames more than 50% of the peptides had sizes above 3 kDa. 

The antihypertensive inhibitions (*I_ACE_*) of FPHs, in terms of percentage values, were always higher than 67% (ranging from 68% to 87%), reaching the best outcome in S_TF. Nevertheless, the differences among samples were not significant (*p* > 0.05). In general, our results were similar and higher than reported for FPHs of wastes from wild fish: board fish [40], hake [41], horse mackerel [42], and Cape fish [43]. The data of bioactivities (*IC_50_*) obtained after dose–response modeling by Weibull equation [27] were also described in Table 6. As in the previous case, these values of angiotensin I-converting enzyme (ACE) inhibiting activity were not significantly different for each type of FPHs. Regarding literature, hydrolysates of thermally defatted salmon backbones performed by Trypsin generated lower activity (*IC_50_* = 0.92 mg FPH/mL) than values presented here [44]. In this context, Carolase PP hydrolysates of salmon trimmings produced after fish mince extraction at different pHs led to similar antihypertensive activity (around 521 μg ACE/mL) [45]. However, a hydrolysate of 300–500 Da obtained from frames of trout, assisted by microwave, showed greater activity (3.6 μg/mL) [46].

## 3. Materials and Methods

### 3.1. Fish Material Processing

Heads, trimmings together with frames of salmonids, rainbow trout (*Oncorhynchus mykiss*), and Atlantic salmon (*Salmon salar*) (Figure S4, Supplementary material), were kindly supplied by a Galician Company (Isidro 1952, S.L., Cambre, A Coruña) that processes gutted Norwegian salmon and grows trout in its farms. These by-products (45–50 kg of each by-product and origin) were frozen and kept at −18 °C until processing. Initially, the 4 types of substrates, heads of rainbow trout (RT_H), trimmings and frames of rainbow trout (RT_TF), heads of salmon (S_H), and trimmings and frames of salmon (S_TF), were ground in a meat mincer (Figure S3E,F, Supplementary material, are examples of by-product minces before hydrolysis). In Table S4 (Supplementary material), the meaning of symbols and abbreviations used in the text are listed.

### 3.2. Optimization of Enzyme Hydrolysis of Salmonid By-Products

Initially, the joint influence of temperature (*T*) and *pH* on the Alcalase 2.4 L (2.4 AnsonUnit/g, AU/g enzyme, Nordisk, Bagsvaerd, Denmark) digestion of RT_H and S_H were evaluated by means of rotatable second order designs (performing 5 replicates in the center of the experimental domain) [47]. Protease concentration, (S:L) ratio, and agitation were maintained constant in these experiments (Table S1, Supplementary material). Maximum hydrolysis (*H_m_*), concentration of soluble protein (Prs), and yield of digestion (V_dig_) were the responses (*Y*) tested. Polynomial equations, relating the effect of independent variables on the responses, were obtained after applying orthogonal least-squares method:

(1) $$Y=b_{0}+\sum_{i=1}^{n}b_{i}X_{i}+\sum_{\underset{j>i}{i=1}}^{n-1}\sum_{j=2}^{n}b_{ij}X_{i}X_{j}+\sum_{i=1}^{n}b_{ii}X_{i}^{2}$$

where: *Y* is the response evaluated, *b_0_* is the constant coefficient, *b_i_* is the coefficient of linear effect, *b_ij_* is the coefficient of combined effect, *b_ii_* is the coefficient of quadratic effect, *n* is the number of variables, and *X_i_* and *X_j_* are the independent variables studied in each case. Student’s t-test (α=0.05) was used to calculate statistical significance of coefficients. Goodness-of-fit was evaluated by means of the coefficients of determination ($R_{}^{2}$) and adjusted coefficients of determination ($R_{adj}^{2}$). Model consistency was established according the values of mean squares ratios from Fisher F test (α = 0.05): *F1* = Model/Total error, being the model acceptable when $F1\geq F_{den}^{num}$; and *F2* = (Model + Lack of fitting)/Model, being the model acceptable when $F2\geq F_{den}^{num}$. $F_{den}^{num}$ are the theoretical values to α = 0.05 with corresponding degrees of freedom for numerator (num) and denominator (den). This set of experiments was performed in a 100 mL glass-reactor configured as a pH-Stat system (with additional control of T, agitation, and reagents addition). After hydrolysis, enzyme deactivation was achieved by heating at 90 °C for 15 min.

On the other hand, the effect of (S:L) ratio on S_H and RT_H hydrolysis was then assessed keeping the other experimental conditions constant in the values of pH and T previously optimized. Similarly, the individual influence of the enzyme concentration on salmonid heads proteolysis was also tested. In all cases, the content of 100 mL-reactors was centrifuged, at the end of hydrolysis period (3 h), for 20 min at 15,000 × g, and the supernatants and sediments (mineral fraction) were quantified.

### 3.3. Production of Enzymatic Hydrolysates from Salmonids By-Products 

Lab-scale hydrolysis was performed in a 5 L glass-reactor (pH-Stat system equipped with additional temperature, agitation, and reagents-addition control), mixing 2 kg of milled by-products in 2 L of distilled water (solid:liquid ratio of (1:1)) employing 5 M NaOH for pH-control. Experimental conditions of hydrolysis were established on the optimal values found in the previous section. At the end of the enzymatic digestion process (3 h), bones were removed by filtration (100 μm) and oils were then recovered by centrifugation (15,000 × g for 20 min) and decantation (for 15 min) from the liquid FPHs. Final hydrolysates were fast warmed (90 °C for 15 min) for Alcalase deactivation.

The hydrolysis degree (*H*, as %) was calculated according to the pH-Stat method [48] and the mathematical models previously reported [22]. The time course of *H* were fitted to the Weibull equation [27]:

(2) $$H=H_{m}\left\{1-\exp\left[-\ln2{\left(\frac{t}{\tau }\right)}^{\beta }\right]\right\}\;\mathrm{with}\;v_{m}=\frac{\beta H_{m}\ln2}{2\tau }$$

where, *H* is the hydrolysis degree (%), *t* is the hydrolysis time (min), *H_m_* is the maximum hydrolysis degree (%), *β* is a dimensionless parameter associated to the slope of the hydrolysis process, *v_m_* is the maximum hydrolysis rate (% min^−1^), and *τ* is the time needed to reach the semi-maximum hydrolysis degree (min). The yield of digestion/liquefaction (V_dig_) of raw material to liquid phase was also calculated (in %) [27].

### 3.4. Chemical and Biological Analyses of Substrates and Bioproducts Obtained

The composition of by-products was obtained by determining: (1) moisture, organic matter, and ash percentage [49]; (2) total protein as total nitrogen × 6.25 [49], and (3) total lipids [50]. The profile of fatty acids from fish oil was quantified by GC-chromatography after chemical methylation [51]. The analyses performed to the hydrolysates were: (1) total sugars [52]; (2) total soluble protein [53]; (3) total protein as total nitrogen × 6.25 [49]; (4) amino acids content by ninhydrin reaction [54], employing an amino acid analyzer (Biochrom 30 series, Biochrom Ltd., Cambridge, UK); and (5) *in vitro* digestibility (pepsin method: AOAC Official Method 971.09) according to the reformulations suggested by Miller et al. [55]. 

Gel Permeation Chromatography (GPC) was used to estimate the molecular weight distributions of FPH. The system (an Agilent 1260 HPLC) was equipped with quaternary pump, injector, column oven, and triple detection (refractive index, diode array, and dual-angle static light scattering). Elution was performed with 0.15 M ammonium acetate/0.2 M acetic acid (pH 4.5) at 1 mL/min. A sample volume of 100 µL was injected onto a set of four Proteema columns (PSS, Germany): precolumn (5 µm, 8 × 50 mm), 30 Å (5 µm, 8 × 300 mm), 100 Å (5 µm, 8 × 300 mm), and 1000 Å (5 µm, 8 × 300 mm) kept at 30 °C. Detectors were calibrated with a polyethylene oxide standard of average number molecular weight 106 kDa (polydispersity index 1.05) from PSS (Germany). Calculations of absolute molecular weights were carried out with refractive index increments (dn/dc) of 0.185. In the case of molecular weight of peptides from FPHs (<10 kDa), the samples of FPHs, after processing by centrifugation on Amicon-10 kDa (MerckMillipore, Germany), were quantified by HPLC (220 nm UV-detection) using Superdex peptide 10/300 GL column (GE Healthcare Life Sciences, UK), with 0.1% trifluoroacetic acid in 30% of acetonitrile as mobile phase (flow rate of 0.4 mL/min) at 25 °C. The standards were Blue Dextran (2 MDa), Cytochrome c (12.4 kDa), Aprotinin (6.5 kDa), Angiotensin II (1046 Da), Leucine encephalin (555 Da), Val-Tyr-Val (379 Da), and Gly-Gln (221 Da).

Antioxidant (AO) and antihypertensive (AH) activities were determined in the hydrolysates by: (a) 1,1-Diphenyl-2-picryhydrazyl (DPPH) radical-scavenging ability using an optimized microplate method [56]; (b) Crocin bleaching assay at microplate scale [57]; (c) ABTS (2,2′-azinobis-(3-ethyl-benzothiazoline-6-sulphonic acid) bleaching method also following a microplate protocol [56]; d) *in vitro* Angiotensin I-converting enzyme (ACE) inhibitory activity (*I_ACE_*) according to the protocol of Estévez et al. [58] and *IC_50_* values (protein-hydrolysate concentration that generates a 50% of maximum *I_ACE_*) were calculated by dose–response modeling [27]. Samples from FPHs for AH and AO measures were prepared at 1 g/L of soluble protein and analysis was done in triplicate.

### 3.5. Numerical and Statistical Analyses

The fitting of experimental data to mathematical equations, with the corresponding estimation of the parameters, was carried out using the nonlinear least-squares (quasi-Newton) method included in the macro ‘Solver’ (Microsoft Excel spreadsheet). “SolverAid” macro was then used to determine the intervals of confidence of parametric estimations (Student’s t test) and the robustness of equations (Fisher’s F test).

## 4. Conclusions

The main experimental conditions of Alcalase 2.4 L proteolysis (pH, T, enzyme concentration, and solid:liquid ratio) were optimized to maximize the production of hydrolysates of salmonids head by-products. Based on those optimal conditions, Alcalase confirmed its high proteolytic capacity producing FPHs of high soluble protein content, a remarkable TEAA/TAA ratio and at least 85% of peptides below 3 kDa. The recovery of fish oils in the same process of FPHs production was also addressed reaching amounts larger than 9% (v/w). Antioxidant activities in FPHs showed values of 56.9% (DPPH) and 16.8 μg of BHT equivalent/mL for S_TF and 9.02 μg of Trolox equivalent/mL for RT_TF. In addition, the data of antihypertensive activities were also relevant to *IC_50_* = 479 μg/mL for S_H. Taking into account the chemical composition of FPHs produced here, they could be incorporated, after drying, in aquaculture feeds as an ingredient for replacing fish meals. Further experiments should be done in this direction to validate this approach and to demonstrate circularity, waste reduction (including life cycle assessment, LCA method), and potential uses of salmonid hydrolysates.

## Figures and Tables

**Table 1:** Chemical composition of salmonids wastes in terms of moisture (Mo), organic matter (OM), and ashes (Ash). Total lipids (Lip), proteins (Pr-tN, as total nitrogen × 6.25), and proteins after degreasing samples (Pr-tN *) were determined using dried substrates. Error bars showed the intervals of confidence for *n* = 3–4 (samples from independent batch) and α = 0.05.

FPHs	Mo (%)	OM (%)	Ash (%)	Lip (%)	Pr-tN (%)	Pr-tN * (%)
**RT_H**	69.6 ± 0.2	27.7 ± 0.2	2.7 ± 0.1	47.7 ± 0.4	35.2 ± 1.0	66.1 ± 3.4
**RT_TF**	66.5 ± 0.9	30.6 ± 0.4	3.0 ± 0.5	44.0 ± 0.9	45.1 ± 2.3	84.5 ± 2.5
**S_H**	62.6 ± 1.0	34.7 ± 1.2	2.7 ± 0.6	54.3 ± 0.3	30.1 ± 1.0	78.3 ± 4.0
**S_TF**	63.1 ± 0.5	34.0 ± 1.0	2.9 ± 0.3	46.2 ± 0.9	44.8 ± 2.0	82.9 ± 3.0

**Table 2:** Polynomial equations describing the combined influence of *pH* and temperature (*T*) on Alcalase proteolysis of RT_H and S_H. Optima values of both independent variables (*T_opt_*, *pH_opt_*) to reach the predicted maximum responses (*Y_max_*) were also calculated.

	Second Order Models	$R_{adj}^{2}$	*T_opt_* (°C)	*pH_opt_*	*Y_max_*
**RT_H**	*H_m_* (%) = 27.10 + 2.72 *pH* – 9.16 *T^2^* – 6.39 *pH^2^*	0.909	55.0	8.30	30.1%
	*V_dig_* (%) = 83.34 – 2.35 *T* + 3.21 *pH* – 12.17 *T^2^* – 6.86 *pH^2^*	0.759	53.3	8.33	83.8%
	*Prs* (g/L) = 55.4 + 4.42 *T* + 3.60 *pH* + 2.25 *T pH* – 8.19 *T^2^* – 13.1 *pH^2^*	0.869	60.2	8.17	56.3 g/L
**S_H**	*H_m_* (%) = 29.44 + 5.30 *T* + 8.54 *pH* – 5.36 *T^2^* – 7.30 *pH^2^*	0.803	63.8	8.83	33.3%
	*V_dig_* (%) = 85.72 + 2.11 T + 8.76 *pH* – 5.62 *T^2^* – 5.62 *pH^2^*	0.779	58.4	9.10	89.3%
	*Prs* (g/L) = 54.71 + 3.49 T + 6.77 *pH* + 3.25 *T pH* – 3.30 *T^2^* – 6.82 *pH^2^*	0.902	70.5	9.00	58.6 g/L

**Table 3:** Kinetic parameters and confidence intervals obtained from Weibull equation modeling the time course of the hydrolysis degree (*H*) of salmonid by-products catalyzed by Alcalase. Determination coefficients of fittings (R^2^) and *p*-values are also shown.

FPHs	*H_m_* (%)	β (Dimensionless)	τ (min)	*v_m_* (% min^−1^)	R^2^	*p*-Values
**RT_H**	29.66 ± 0.17	0.801 ± 0.008	32.07 ± 0.37	0.257 ± 0.004	0.999	<0.005
**RT_TF**	30.94 ± 0.25	0.807 ± 0.011	34.19 ± 0.56	0.253 ± 0.005	0.999	<0.005
**S_H**	31.55 ± 0.06	0.770 ± 0.006	15.85 ± 0.11	0.531 ± 0.004	1.000	<0.005
**S_TF**	34.27 ± 0.10	0.756 ± 0.007	18.41 ± 0.14	0.488 ± 0.005	0.999	<0.005

**Table 4:** Proximate analysis and mass balances of the products generated by Alcalase proteolysis of salmonid by-products. Errors are the intervals of confidence for *n* = 18–20 (replicates of independent hydrolysates) and α = 0.05. m_b_: bones recovered (%); V_oil_: oil isolated (%); V_dig_: yield of substrate digestion (%); Prs: Total soluble protein; TS: Total sugars; Dig: *in vitro* Digestibility; Pr-tN: Total protein determined as total nitrogen × 6.25.

FPHs	m_b_ (%)	V_oil_ (%)	V_dig_ (%)	Prs (g/L)	Pr-tN (g/L)	TS (g/L)	Dig (%)
**RT_H**	9.98 ± 1.31	9.36 ± 0.75	88.4 ± 1.2	47.8 ± 4.8	53.1 ± 1.9	1.40 ± 0.10	92.5 ± 3.2
**RT_TF**	9.43 ± 0.52	10.63 ± 0.42	84.4 ± 1.1	53.9 ± 5.1	58.4 ± 2.7	1.22 ± 0.10	93.2 ± 2.5
**S_H**	11.13 ± 1.36	11.37 ± 0.60	89.8 ± 0.7	61.0 ± 1.3	64.2 ± 3.1	1.29 ± 0.09	93.0 ± 2.2
**S_TF**	11.59 ± 0.44	9.30 ± 0.12	86.3 ± 1.1	69.7 ± 2.1	71.1 ± 2.6	1.50 ± 0.10	94.1 ± 2.8

**Table 5:** Average molecular weights (as Mn and Mw) and associated confidence intervals for *n* = 5 (samples from independent hydrolysates) and α = 0.05. Percentage of peptides distribution between molecular weight ranges was also determined. PDI: polydispersity index.

FPHs	Mn (Da)	Mw (Da)	PD	0–0.2 kDa (%)	0.2–0.5 kDa (%)	0.5–1 kDa (%)	1–3 kDa (%)	>3 kDa (%)
**RT_H**	920 ± 110	1944 ± 264	2.11	13.7 ± 1.0	8.4 ± 0.5	25.9 ± 1.4	38.0 ± 3.9	14.0 ± 0.5
**RT_TF**	1067 ± 152	1682 ± 65	1.58	8.4 ± 1.3	8.5 ± 0.3	25.1 ± 0.9	47.6 ± 8.1	10.4 ± 0.2
**S_H**	1235 ± 91	1945 ± 136	1.57	8.0 ± 2.2	7.8 ± 0.9	17.2 ± 3.6	52.0 ± 3.9	15.0 ± 0.4
**S_TF**	944 ± 40	1442 ± 51	1.53	7.8 ± 1.1	16.0 ± 1.3	24.6 ± 1.2	43.3 ± 2.3	8.3 ± 0.1

**Table 6:** Bioactivities of FPHs from salmonids wastes. Errors show the intervals of confidence for *n* = 9 (α = 0.05).

FPHs	AO			AH	
	DPPH (%)	ABTS (μg BHT/mL)	Crocin (μg Trolox/mL)	*I_ACE_* (%)	*IC_50_* (μg Protein/mL)
**RT_H**	48.22 ± 1.34	14.98 ± 0.51	8.71 ± 0.24	82.1 ± 23.0	508.9 ± 58.6
**RT_TF**	53.22 ± 1.68	15.12 ± 0.24	9.02 ± 0.39	67.8 ± 10.7	975.4 ± 476.6
**S_H**	45.25 ± 2.89	13.12 ± 1.01	7.52 ± 0.09	71.9 ± 15.8	478.5 ± 178.6
**S_TF**	56.85 ± 3.10	16.77 ± 1.39	8.45 ± 0.87	87.0 ± 19.0	653.7 ± 158.3

## Supplementary Materials

The following are available online at https://www.mdpi.com/1660-3397/17/12/676/s1, Table S1: Experimental design for the optimization of the FPHs, Table S2: Fatty acids content from fish oils recovered from different by-products of salmonids, Table S3: Amino acids content of FPHs produced from salmonids by-products, Table S4: List of symbols and abbreviations used in the text, Figure S1: Flowchart of fish discards valorization, Figure S2: Pictures describing the production of salmonids hydrolysates, Figure S3: Pictures of the salmonids by-products (raw materials).
