# Supplementary material for: Valorization of Aquaculture By-Products of Salmonids to Produce Enzymatic Hydrolysates: Process Optimization, Chemical Characterization and Evaluation of Bioactives
Source: Mar Drugs. 2019 Nov 30;17(12):676. doi: 10.3390/md17120676 (PMC6950744; doi:10.3390/md17120676)
Supplement: Supplementary file 1 [file marinedrugs-17-00676-s001.pdf]

Valorization of Aquaculture by-Products of Salmonids to Produce Enzymatic Hydrolysates: Process Optimization, Chemical Characterization and Evaluation of Bioactives

SUPPLEMENTARY MATERIAL

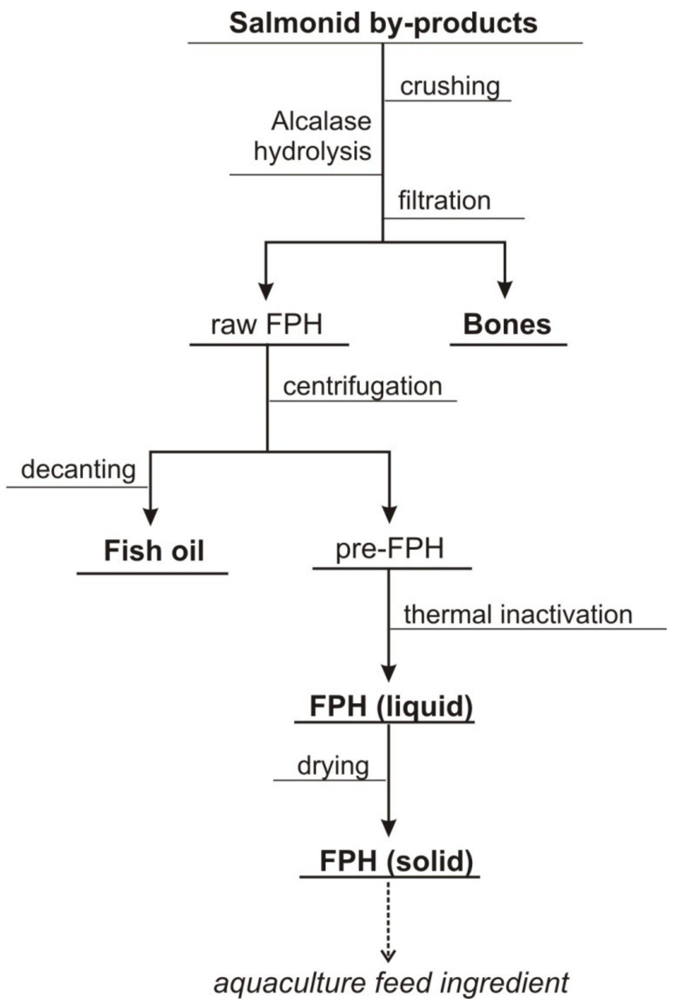

Figure S1. Schematic flowchart of by-products processed through enzymatic hydrolysis.

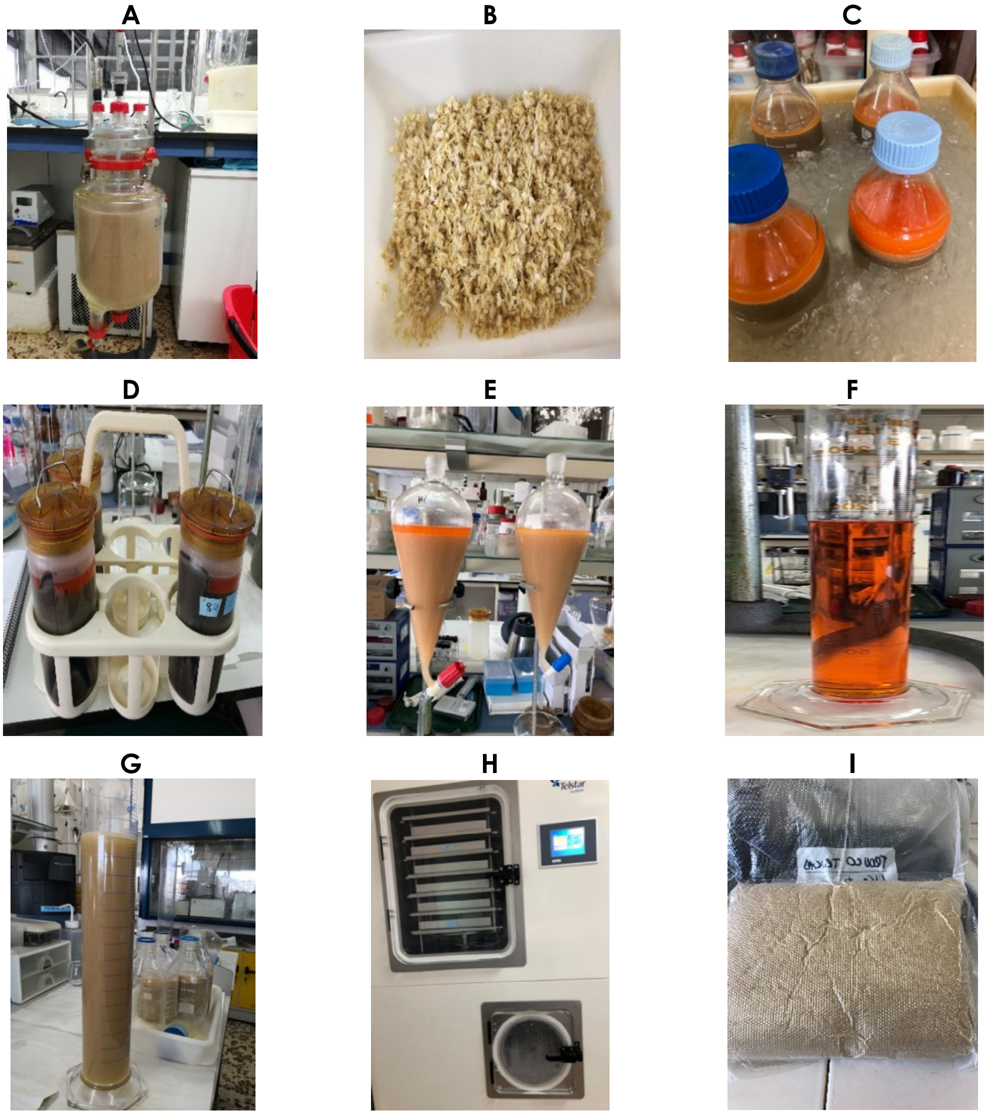

Figure S2. Different sequences of enzymatic hydrolysis of salmonid wastes in a 5L-pH-stat reactor (A) with the differential recovery of clean bones (B) and fish oils (F) together with the production of liquid (G) and dried FPHs (I) by means of a freeze-drying equipment (H). The rest of the images show the cooling of hydrolysates (C) prior centrifugation (D) and decantation of FPHs to separate fish oils (E).

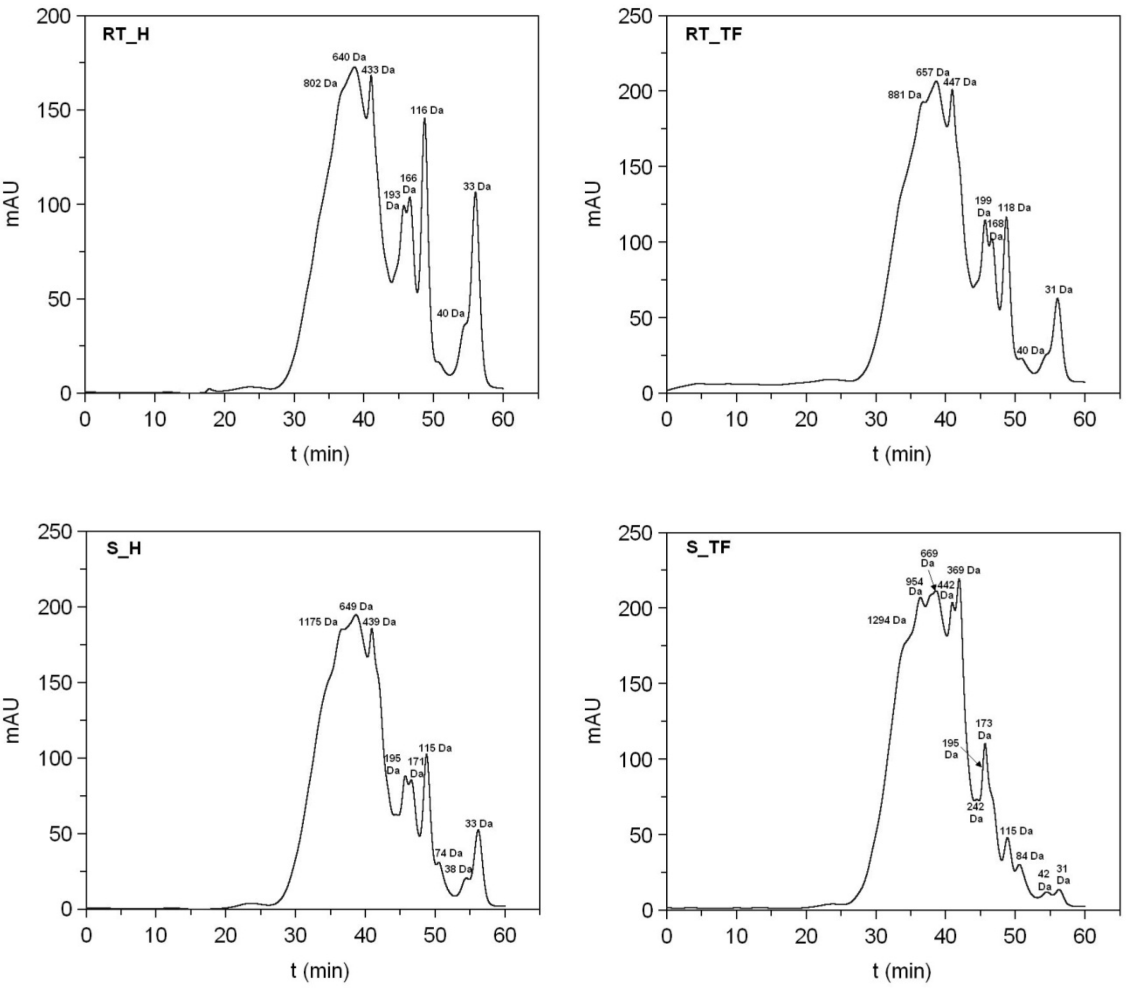

Figure S3. Size exclusion chromatographic profiles of salmonid hydrolysates from Superdex peptide 10/300 GL column, elution phase: 0.1% trifluoroacetic acid in 30% of acetonitrile, flow rate: 0.4 mL/min at 25ºC, detection of UV: 210 nm.

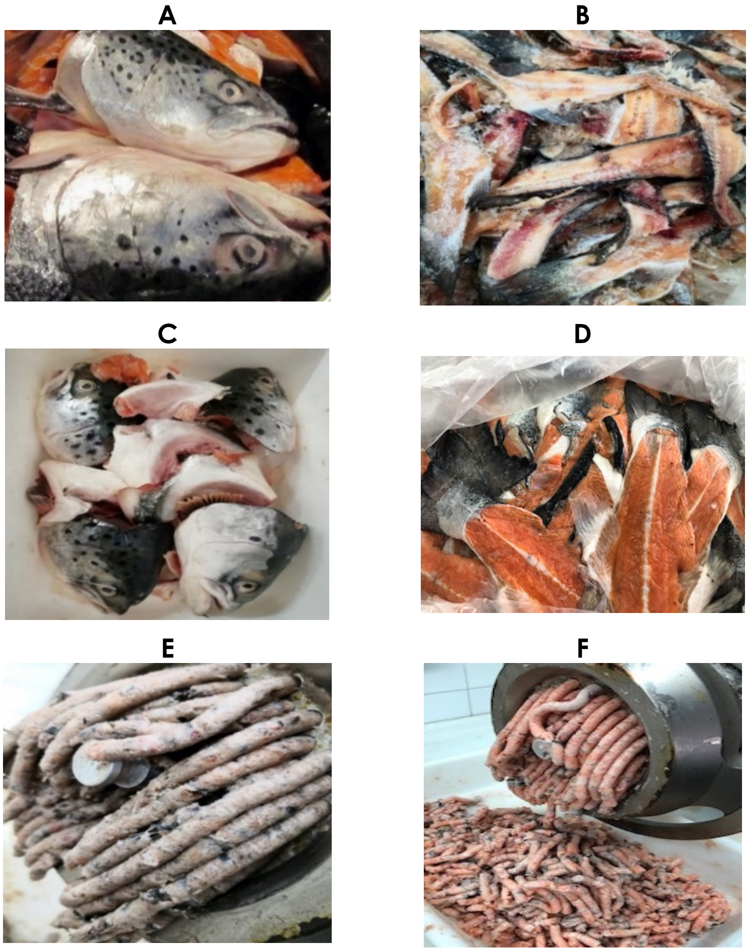

Figure S4. Pictures of the salmonids by-products processed in the IIM-CSIC: A) heads of rainbow trout, B) trimmings and frames of rainbow trout, C) heads of salmon and D) trimmings and frames of salmon. E) minced of rainbow trout by-products and F) minced of salmon by-products.

Table S1. Experimental domain and coding of the independent variables in the factorial design executed to study the joint effect of pH and temperature on the Alcalase hydrolysis of salmonid by-heads.

	Natural values	
**Coded values**	**pH**	**T (ºC)**
-1.41	6.0	30.0
-1	6.6	37.3
0	8.0	55.0
+1	9.4	72.7
+1.41	10.0	80.0

Codification: $V_c = (V_n - V_0)/\Delta V_n$
Decodification: $V_n = V_0 + (\Delta V_n \times V_c)$
V_n = natural value of the variable to codify
ΔV_n = increment of V_n for unit of V_c
V_0 = natural value in the centre of the domain
V_c = codified value of the variable

Constant conditions
Agitation= 200 rpm; r (S:L)= 1:1; [Alcalase]= 0.5% (v/w) or 12 AU/kg of heads, time of hydrolysis= 3 h.

Table S2. Fatty acids content (as % of total fatty acids) in the fish oils recovered from RT_H, RT_TF, S_H and S_TF, complementary to the production of FPHs. Errors are the confidence intervals for n=6 (samples from independent hydrolysates) and α=0.05.

Formula	Fatty acids	RT_H	RT_TF	S_H	S_TF
**C8:0**	Caprylic acid	0.03±0.03	-	-	-
**C10:0**	Capric acid	0.07±0.01	-	-	-
**C12:0**	Lauric acid	0.12±0.01	-	-	-
**C13:0**	Tridecanoic acid	0.06±0.01	-	-	-
**C14:0**	Myristic acid	0.74±0.01	0.69±0.01	2.23±0.25	2.29±0.16
**C14:1**	Myristoleic acid	0.10±0.03	0.01±0.00	-	-
**C15:0**	Pentadecanoic acid	0.13±0.01	0.05±0.01	-	-
**C15:1**	Pentadecenoic acid	4.10±0.11	4.42±0.05	-	-
**C16:0**	Palmitic acid	6.13±0.09	6.58±0.03	7.49±0.82	6.65±0.73
**C16:1n7c**	Palmitoleic acid	2.77±0.18	3.49±0.17	2.09±0.24	2.06±0.67
**C17:0**	Heptadecanoic acid	0.17±0.09	0.06±0.00	-	-
**C17:1**	Heptadecanoleic acid	0.69±0.51	0.07±0.01	-	-
**C18:0**	Stearic acid	1.80±0.05	1.87±0.02	2.13±0.11	2.07±0.17
**C18:1n9c,t**	Oleic acid	52.85±0.46	57.48±0.16	44.56±1.31	40.90±2.20
**C18:2n6c,t**	Linoleic acid	12.06±0.27	13.19±0.14	22.66±2.20	22.31±1.42
**C20:0**	Arachidic acid	0.42±0.22	0.09±0.02	-	-
**C18:3n6**	γ-Linolenic acid	0.31±0.04	0.24±0.00	-	-
**C18:3n3**	Linolenic acid	2.04±0.06	2.13±0.03	5.83±0.85	6.79±1.68
**C20:1n9**	Eicosenoic acid	1.88±0.68	2.23±0.27	6.41±2.21	10.52±3.06
**C21:0**	Henicosanoic acid	0.50±0.01	0.50±0.03	-	-
**C20:2n6**	Eicosadienoic acid	0.51±0.07	0.95±0.15	1.01±0.20	1.16±0.08
**C22:0**	Docosanoic acid	0.16±0.02	0.06±0.01	-	-
**C20:3n6**	Dihomo-linolenic acid (DGLA)	0.32±0.04	0.30±0.03	-	-
**C20:4n6**	Arachidonic acid	0.17±0.08	0.22±0.01	-	-
**C23:0**	Tricosanoic acid	0.11±0.01	0.03±0.01	-	-
**C21:4n3**	Heneicosatetraenoic acid	1.86±0.14	0.59±0.09	1.88±0.36	2.09±0.18
**C22:2n6**	Docosadienoic acid	0.18±0.13	0.01±0.00	-	-
**C20:5n3**	Eicosapentaenoic acid (EPA)	0.63±0.04	0.55±0.02	0.34±0.08	0.38±0.05
**C24:0**	Lignoceric acid	0.26±0.02	-	-	-
**C24:1n9**	Nervonic acid	6.72±0.74	1.95±0.80	-	-
**C22:6n3**	Docosahexaenoic acid (DHA)	2.14±0.09	2.27±0.07	3.36±0.23	2.78±0.45
	**DHA+EPA (%)**	2.77±0.13	2.82±0.08	3.70±0.27	3.16±0.45
	**r: ω-3 / ω-6**	0.49±0.01	0.37±0.01	0.49±0.06	0.51±0.06

Table S3. Amino acids content of FPH (% or g/100 g total amino acids) from salmonid by-products. OHPro: hydroxyproline. Pr: protein concentration calculated. in g/L. as the total sum of amino acids present in FPH. TEAA/TAA: ratio total essential amino acids for human/total amino acids. Errors are the confidence intervals for n=16-20 (replicates of independent hydrolysates) and α=0.05.

Amino acids	RT_H	RT_TF	S_H	S_TF
**Asp**	9.78±0.19	10.32 ±0.20	9.61±0.30	10.33±0.06
**Thr**	4.38±0.22	4.44±0.15	3.83±0.39	2.95±0.04
**Ser**	5.00±0.20	4.83±0.06	4.98±0.05	4.98±0.11
**Glu**	13.89±0.14	14.98±0.34	13.42±0.45	13.23±0.08
**Gly**	9.93±1.10	8.94±2.94	12.49±1.11	11.08±0.27
**Ala**	7.19±0.31	6.98±0.22	7.92±0.45	8.45±0.03
**Cys**	0.76±0.09	0.74±0.05	0.75±0.11	0.83±0.03
**Val**	4.35±0.33	4.24±0.22	3.39±0.16	3.44±0.30
**Met**	3.16±0.10	3.33±0.15	3.13±0.28	3.82±0.33
**Ile**	3.22±0.29	3.21±0.23	2.28±0.22	2.02±0.16
**Leu**	7.09±0.33	7.19±0.07	6.17±0.33	6.36±0.22
**Tyr**	3.36±0.18	3.39±0.22	3.37±0.45	4.40±0.17
**Phe**	4.38±0.25	4.09±0.15	4.93±0.89	7.15±1.13
**His**	2.20±0.49	2.18±0.02	2.00±0.13	2.11±0.17
**Lys**	7.78±0.42	8.60±0.13	7.04±0.46	7.96±0.37
**Arg**	5.97±0.10	5.96±0.14	5.69±0.40	4.44±0.03
**OHPro**	2.25±0.37	1.86±0.38	2.85±0.62	2.00±0.14
**Pro**	5.30±0.34	4.72±0.16	6.15±0.83	4.45±0.28
**Pr (Σaa) (g/L)**	51.96±2.83	66.08±1.72	66.08±3.72	72.71±2.13
**TEAA/TAA (%)**	36.56	37.24	32.77	35.81

Table S4. List of symbols and abbreviations used in the text.

**RT_H:**	Heads of rainbow trout	**TEAA:**	Total essential amino acids
**RT_TF:**	Trimmings + frames of rainbow trout	**TAA:**	Total amino acids
**S_H:**	Heads of salmon	**DHA:**	Docosahexaenoic acid
**S_TF:**	Trimmings + frames of salmon	**EPA:**	Eicosapentaenoic acid
**Mo:**	Moisture	**Mn:**	Number average molecular weight
**OM:**	Organic matter	**Mw:**	Average molecular weight
**Ash:**	Ashes	**PD:**	Index of polydispersity
**Lip:**	Total lipids	$\mathbf{V_{dig}}$**:**	Yield of substrate digestion
**Pr-tN:**	Total protein as total nitrogen x 6.25	***H*:**	Degree of hydrolysis
**Pr-tN*:**	Total protein after degreasing	H_m**:**	Maximum degree of hydrolysis
**FPH:**	Fish protein hydrolysate	v_m**:**	Maximum hydrolysis rate
T_{opt}**:**	Optimum temperature	τ**:**	Time needed to reach the semi-maximum value of *H*
pH_{opt}**:**	Optimum pH	β**:**	Dimensionless parameter
**UV:**	Ultraviolet wavelength	***Y*:**	Predicted response
$\mathbf{m_b}$**:**	Percentage of bones recovered	Y_{max}**:**	Predicted maximum response
$\mathbf{V_{oil}}$**:**	Percentage of oil isolated	**Prs:**	Total soluble protein
**TS:**	Total sugars	**Pr (Σaa):**	Total protein as sum of amino acids
**Dig:**	*In vitro* digestibility	**AO:**	Antioxidant activity
**S:L:**	Solid:liquid ratio	**AH:**	Antihypertensive activity
**DPPH:**	1,1-Diphenyl-2-picryhydrazyl	I_{ACE}**:**	ACE inhibitory activity
**ABTS:**	2,2′-azinobis-(3-ethyl-benzothiazoline-6-sulphonic acid	IC_{50}**:**	FPH concentration that generates 50% of maximum I_{ACE}.
